# Psychiatric Presentation of Wilson’s Disease: A Rare Disease With an Unusual Manifestation

**DOI:** 10.7759/cureus.81645

**Published:** 2025-04-03

**Authors:** Inês Baptista, Patrick Alves

**Affiliations:** 1 Psychiatry, Centro Hospitalar São João, Porto, PRT

**Keywords:** claustrum sign, liaison psychiatry, secondary mood syndrome, suicide risk, wilson disease neuropsychiatric manifestation

## Abstract

Wilson’s disease is a rare disorder characterized by the accumulation of copper in multiple organs. Psychiatric symptoms are somewhat common but can take many different forms, which complicates their identification. We present a case where suicidal ideation and severe sleep disturbance were the central symptoms of the initial presentation. This case shows how psychiatric symptoms in uncompensated Wilson’s disease can look like and how they are managed, as well as a psychological perspective into therapy nonadherence in chronic illness.

## Introduction

Wilson’s disease is a rare autosomal recessive disorder characterized by an impairment of the biliary excretion of copper, resulting in its accumulation in multiple tissues, including the liver, brain, and cornea [1]. Wilson’s disease has an estimated prevalence of about 30 cases per million, and it appears to affect women and men equally. Most patients develop symptoms between the ages of 3 and 55 years, with a predominance in adolescents and young adults [1,2].

Hepatic dysfunction is the initial manifestation of the disease in about 40-50% of individuals, and it can assume multiple forms. This includes varying manifestations ranging from acute transient hepatitis to an asymptomatic elevation of liver enzymes [1]. Neurologic impairment is also a common presentation (40-60% of individuals), indicating that copper accumulation in the brain occurs early in the disease. Symptoms include multiple types of tremors, dysarthria, dystonia, parkinsonism, choreoathetosis, cerebellar dysfunction, and ataxia [3].

Psychiatric symptoms are common and can be present in the initial presentation in 20-65% of patients or develop years after the establishment of the disease. Most psychiatric manifestations are mood disorders (either depression or mania), but personality changes, sleep disturbances, psychosis, and cognitive changes are also prevalent. These frequently arise associated with neurologic dysfunction, which reflects the common origin of both in the central nervous system [4-6].

Despite the various targets of damage of this disease, there are treatments available for patients. The first-line medications include chelating agents and zinc. Non-responders or patients who present with severe liver damage may need more aggressive strategies, including a liver transplant [7].

Wilson’s disease, while rare, frequently manifests with diverse and complex psychiatric symptoms, especially when it presents as an initial or unexpected occurrence in an otherwise stable individual. The rarity with which we come across these types of cases and their special nature underscores the critical challenges associated with such manifestations and offers insights into the management strategies for these cases.

In this case report, we present a patient diagnosed with Wilson’s disease in young childhood who started to develop neuropsychiatric symptoms after stopping his medication. The context of his nonadherence and the symptoms he manifested afterwards are the central points of psychiatric relevance.

## Case presentation

A 29-year-old patient, of white ethnicity, married, father of a 13-month-old girl, and logistics worker in the event industry, was assessed in the emergency department while accompanied by his wife on the fifth of January 2024 following an urgent referral from his family doctor who evaluated the patient earlier that day. This assessment stated severe difficulties sleeping and suicidal ideation. He was already on medical leave in the previous month, initially, the last week of November, because of a viral respiratory illness, which continued through December 2023 due to complaints of sialorrhea, difficulties with concentration, and paresthesia.

Since he became a father in December 2022, he started to have trouble sleeping in the first semester because his daughter had rough nights, crying for most of the time, and later because he felt anxious about something being wrong with his child. These sleep problems became gradually worse, culminating in almost complete insomnia in the couple of months preceding this evaluation. The patient expressed that he started to take less care of himself as he felt that he had no energy to complete those tasks. He also described feeling sluggish, and like his body struggled to react to his thoughts and actions, making it impossible for him to take care of and play with his daughter. This brought serious distress to the patient, who stated that during the sleepless nights he had “bad thoughts” of killing himself by throwing himself onto a train track near his house to ease his suffering. These thoughts were passive and sparse in the first months, but became constant and gradually more structured in the two weeks before the trip to the emergency room. He was described by the emergency psychiatrists as severely depressed with anhedonia and avolition, slowed down movements and speech, as well as low affective reactivity. There were no ascertainable psychotic symptoms. Physical examination and vital signs were normal. Neurological examination was not performed in the emergency room.

According to his relevant medical history, the patient had Wilson’s disease, diagnosed in childhood and medicated with zinc, which was then substituted with trientine at nine years old. The patient then remained asymptomatic through adulthood. He had a history of an anxiety disorder in his late teenage years, which remitted a few months after the introduction of 5 mg of Escitalopram daily. This medication was stopped after nine months with no recrudescence of symptoms. The patient denied relevant family history, psychiatric or otherwise. Regarding family structure, the patient was an only child, and his parents were very present in his and his daughter’s lives. Due to the risk of suicide in a severely depressed patient, a psychiatric hospitalization was proposed and accepted by the patient.

Following hospital admission, antidepressant therapy with sertraline 50 mg daily was initiated, along with diazepam 5 mg three times a day for anxiety, quetiapine modified-release 50 mg at dinner, and trazodone 100 mg and lorazepam 2.5 mg at bedtime. In a subsequent interview, the patient revealed that he had not been taking his Wilson’s disease medication (trientine, 600 mg twice a day), a fact already stated in a gastroenterology appointment in early December, when the symptoms became evident. The patient described taking it irregularly since his daughter was born in December 2022 and stopping it completely around July of the following year. This had already triggered concerns about neurological involvement, which led to a neurology appointment in February 2024.

Due to the priority of treating the organic consequences, after five days of hospitalization in psychiatry and subsidence of suicidal ideation, the patient was transferred to São João Hospital Centre to be assessed by gastroenterology, which admitted the patient to its department. Here, he was evaluated and treated by liaison psychiatry, with two visits. In the first visit, the antidepressant was switched to fluoxetine 20 mg one time a day, diazepam and quetiapine were stopped, and sedative medications such as trazodone or lorazepam were kept only at night to treat the severe insomnia, while avoiding increased sluggishness during the day. In the second visit, on day 12 of hospitalization, the patient was reevaluated, and no therapeutic adjustments were made as he had already presented an improvement in mood and energy, as well as maintaining a reasonable sleep quality and duration. At this time, the patient did not manifest suicidal ideation, neither spontaneously nor when questioned, and started to tell us about his daughter with delight, and spoke about future plans with his family.

**Figure 1:**
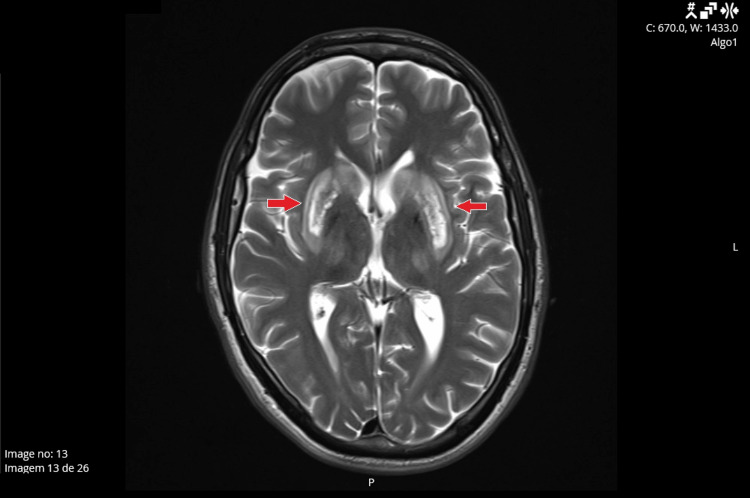
Brain MRI. Axial cuts of the T2 sequence of the patient’s brain MRI with contrast in January 2024 demonstrate a hypersignal and edema bilaterally in the putamen and caudate nuclei, along with a hypersignal on the globus pallidus, translating likely to copper deposits. The arrows demonstrate the “bright claustrum sign” in this patient’s image, which is attributed to a hypersignal of the claustrum.

During this hospitalization, he was also subjected to a brain MRI (Figure 1). The image showed significant and bilateral central nervous system involvement, with copper deposits on globus pallidus and edema of the putamen and caudate nuclei, translating to a “bright claustrum sign.” Hepatic imaging and elastography showed significant fibrosis, and bloodwork showed elevated transaminases, with no other findings. Due to severe hepatic involvement, the patient was put on the hepatic transplant waiting list.

After 22 days of hospitalization, the patient presented a remission from suicidal ideation and an improvement in sleep quality and duration, as the patient started to take less time to fall asleep and awoke less frequently during the night. He also presented a gradual but incomplete recovery from the depressive symptoms at discharge, some relief from feelings of guilt and hopelessness, but maintained important functional impairment caused by the fatigue and avolition. The psychiatric medication was maintained on an outpatient basis, and the patient was referred to a liaison psychiatry consultation. He also maintained dysarthria, dysphagia, slow movements and speech, as well as a sluggish sensation. These neurological symptoms continued in the months following hospital discharge, along with the emergence of fine postural tremors of both hands. To better manage and begin rehabilitation for these symptoms, the patient took speech therapy sessions, focused on dysarthria and dysphagia.

In August 2024, the patient was submitted to a hepatic transplant, which occurred with no immediate complications. The patient maintained chelating therapy with trientine. Follow-ups in neurology showed that the symptoms continued, but a small but significant improvement was observed when compared to the state before the transplant. An incomplete remission is expected as the brain damage shown in radiological tests is potentially irreversible.

Follow-ups with psychiatry were performed remotely due to the patient’s fragile state and distance from the hospital. The medications were not changed in the first months. The patient’s remission from the severe insomnia was maintained at home, but other depressive symptoms continued, mainly feelings of sadness and avolition, which are now partly caused by the patient’s post-transplant status.

## Discussion

In this case, a patient with Wilson’s disease presented a severe depressive episode with persistent insomnia and suicidal ideation. When the previous history was explored, it became evident that the patient had not been taking the prescribed trientine for at least the last four to five months [1]. This seemed to have occurred in the context of a lack of self-care and insomnia presented by the patient at the time of becoming a new father to his daughter. Despite the patient identifying fatherhood as a positive and desired step in his life, it appears that it triggered a chain of events culminating in this hospital admission and its consequences.

The existing psychiatric symptomatology can easily fit a severe depressive episode with suicidal ideation. The patient presented with low energy, anhedonia, avolition, and feelings of sadness. He also had a severe sleep disturbance with multiple nights spent awake. Suicidal ideation can also manifest in these cases, and it was coupled with low self-esteem, with thoughts of worthlessness and hopelessness [8]. These were all evident when a psychiatric evaluation was made, hence the need for behavioral containment in the context of a hospital admission.

However, a more likely diagnosis for this patient was an organic mood disorder, with depressive characteristics, caused by uncompensated Wilson’s disease. These very severe symptoms presented rather quickly, in just a couple of weeks, which is rare in a severe depressive episode, where patients usually get gradually worse, presenting depressive symptoms for several months before this severity is attained. There is also a very evident causal and temporal link between the abandonment of medication and the beginning of these symptoms, as well as a clear central nervous system involvement in an MRI. Additionally, severe sleep disturbances and mood impact are one of the most common psychiatric symptoms associated with uncompensated Wilson’s disease [4-6]. Personality and behavioral shifts are also well described in patients with Wilson’s disease: patients lower their performance at work and home tasks, as well as becoming more irritable and enjoying things less [9,10]. These were symptoms shown by the patient, which he, ultimately, described as getting him to the point of suicidal ideation. This is also a common symptom in these patients, occurring in up to 16% of those affected, with no close association to other symptoms [11].

The definitive diagnosis is complicated by the fact that neither the reintroduction of the chelating medication, as a means of removing the present copper, nor a hepatic transplant, by establishing a normal copper metabolism, will necessarily improve the neuropsychiatric symptoms in the short term. This is a result of the irreversibility of the central nervous system damage, which was observed in this patient [1]. However, at least a slight recovery is expected when accounting for various factors. We need to remove the aggressor, copper accumulation, by reinstating the treatment (medications and, ultimately, a liver transplant). We should introduce antidepressant therapies to improve symptomatology. Lastly, hope for brain plasticity, which can have a role in the loss of function caused by brain injury.

This case highlights the importance of therapeutic adherence. The patient in question was asymptomatic before this episode, which is the population that abandons medication more frequently in Wilson’s disease [12,13]. Although this patient did not have many logistical hurdles to acquire the medications, a chronic treatment can pose an inconvenience, especially when patients have unpredictable schedules, as an event industry worker would, or have major life changes, like when the patient became a father. It has also been shown that lower levels of general education and disease awareness are correlated with lower levels of adherence, which could also shed some light on the factors that led to this case [14].

Despite a hepatic transplant, the patient continues to experience some neuropsychiatric symptoms due to irreversible brain damage. Future management should focus on stabilizing medications for depression and neurological rehabilitation to support recovery and improve quality of life. This case underscores the importance of adherence to medications in chronic conditions and the need for long-term multidisciplinary management of these patients.

## Conclusions

Wilson’s disease patients must adhere to their treatment for their entire lives, which can pose different challenges for both them and their medical team. Active disease can present with a very wide range of symptoms, including those of a psychiatric nature. Although psychiatric manifestations are common, it is rare for clinicians to recognize and focus on them, making it challenging to diagnose Wilson’s disease based solely on psychiatric symptoms and difficult to manage these manifestations. Only a careful and thorough review of the patient’s history and other symptoms can highlight the role of Wilson’s disease in the mental well-being of these patients.

## References

[1] Poujois A, Woimant F (2018). Wilson's disease: a 2017 update. Clin Res Hepatol Gastroenterol.

[2] Taly AB, Meenakshi-Sundaram S, Sinha S, Swamy HS, Arunodaya GR (2007). Wilson disease: description of 282 patients evaluated over 3 decades. Medicine (Baltimore).

[3] Noureen N, Rana MT (2011). Neurological Wilson disease in children: a three years experience from Multan. J Pak Med Assoc.

[4] Svetel M, Potrebić A, Pekmezović T (2009). Neuropsychiatric aspects of treated Wilson's disease. Parkinsonism Relat Disord.

[5] Shanmugiah A, Sinha S, Taly AB (2008). Psychiatric manifestations in Wilson's disease: a cross-sectional analysis. J Neuropsychiatry Clin Neurosci.

[6] Zimbrean PC, Schilsky ML (2014). Psychiatric aspects of Wilson disease: a review. Gen Hosp Psychiatry.

[7] Seetharaman J, Sarma MS (2021). Chelation therapy in liver diseases of childhood: current status and response. World J Hepatol.

[8] American Psychiatric Association (2022). Diagnostic and statistical manual of mental disorders: DSM-5-TR.

[9] Brewer GJ (2005). Behavioral abnormalities in Wilson's disease. Adv Neurol.

[10] Akil M, Brewer GJ (1995). Psychiatric and behavioral abnormalities in Wilson's disease. Adv Neurol.

[11] Oder W, Grimm G, Kollegger H, Ferenci P, Schneider B, Deecke L (1991). Neurological and neuropsychiatric spectrum of Wilson's disease: a prospective study of 45 cases. J Neurol.

[12] Jacquelet E, Poujois A, Pheulpin MC, Demain A, Tinant N, Gastellier N, Woimant F (2021). Adherence to treatment, a challenge even in treatable metabolic rare diseases: a cross sectional study of Wilson's disease. J Inherit Metab Dis.

[13] Masełbas W, Członkowska A, Litwin T, Niewada M (2019). Persistence with treatment for Wilson disease: a retrospective study. BMC Neurol.

[14] Maselbas W, Litwin T, Czlonkowska A (2019). Social and demographic characteristics of a Polish cohort with Wilson disease and the impact of treatment persistence. Orphanet J Rare Dis.

